# Effects of paclobutrazol seed priming on seedling quality, photosynthesis, and physiological characteristics of fragrant rice

**DOI:** 10.1186/s12870-023-04683-0

**Published:** 2024-01-17

**Authors:** Yingying Zhang, Zhenzhen He, Pipeng Xing, Haowen Luo, Zhuosheng Yan, Xiangru Tang

**Affiliations:** 1 State Key Laboratory for Conservation and Utilization of Subtropical Agro-bioresources, College of Agriculture, South China Agricultural University, Guangzhou, 510642 China; 2 Scientific Observing and Experimental Station of Crop Cultivation in South China, Ministry of Agriculture and Rural Affairs, Guangzhou, 510642 China; 3 Guangzhou Key Laboratory for Science and Technology of Fragrant rice, Guangzhou, 510642 China; 4 Guangzhou Golden Rice Agricultral Science and Technology Co, Ltd, Guangzhou, 510900 China

**Keywords:** 2-acetyl-1-pyrroline, *BADH2*, Gene expression

## Abstract

**Background:**

Paclobutrazol is widely used in the agricultural field. This study investigated the effects of seed priming with different concentrations of paclobutrazol on seedling quality, 2-acetyl-1-pyrroline (2-AP, a key aroma component of fragrant rice) biosynthesis, and related physiological and biochemical indicators in fragrant rice seedlings.

**Results:**

The experiment is being conducted at the College of Agriculture, South China Agricultural University. In the experiment, three concentrations of paclobutrazol (Pac 1: 20 mg·L^−1^; Pac 2: 40 mg·L^−1^; Pac 3: 80 mg·L^−1^) were used to initiate the treatment of fragrant rice seeds, while water treatment was used as a control (CK). The results showed that compared with CK, paclobutrazol treatment reduced plant height, increased stem diameter, and increased fresh and dry weight of aromatic rice seedlings. Moreover, paclobutrazol treatment also increased the seedlings’ photosynthetic pigment content and net photosynthetic rate.

**Conclusions:**

This study demonstrates that paclobutrazol primarily increases the content of proline by reducing the content of glutamate and down-regulating the expression of *P5CS2*, thereby promoting the conversion of proline to the aromatic substance 2-AP. Under the appropriate concentration of paclobutrazol (40 mg·L^−1^~80 mg·L^−1^), the seedling quality, stress resistance, and aroma of fragrant rice can be improved.

**Supplementary Information:**

The online version contains supplementary material available at 10.1186/s12870-023-04683-0.

## Background

**Fig. 1:**
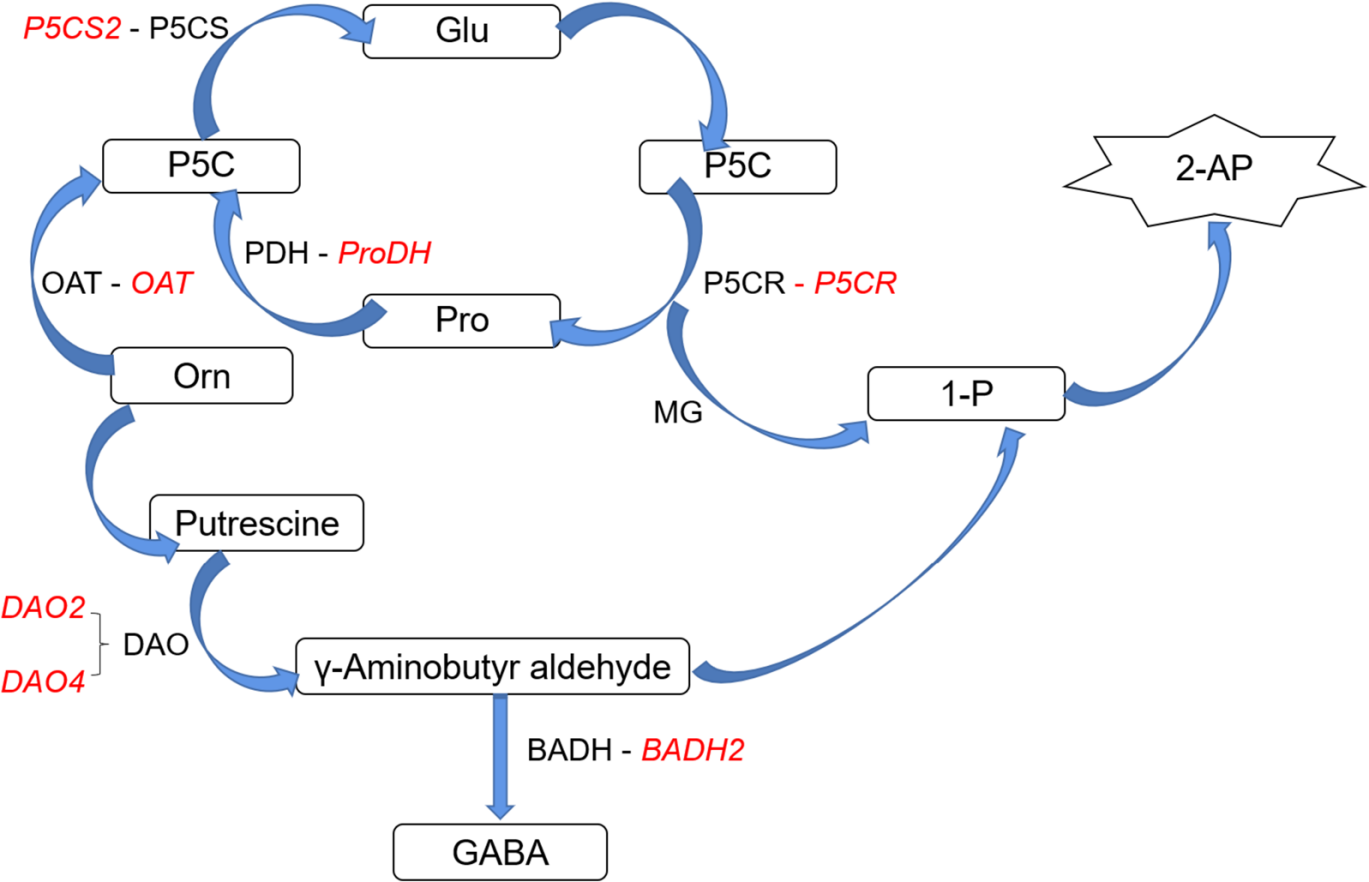
The schematic representation of 2-AP biosynthesis pathway in fragrant rice. The gene names that regulate the relevant enzymes are highlighted in red

With the continuous development of the world economy, the demand for high-quality rice is also increasing [1]. Fragrant rice (*Oryza sativa* L.), a high-quality rice with a unique aroma, is increasingly favored by consumers [2]. The reasons for the aroma formation of fragrant rice are complex and involve multiple compounds [3]. According to relevant studies, fragrant rice grains contain about 300 volatile compounds [4]. Still, among many compounds, 2-acetyl-1-pyrroline (2-AP) is considered the essential compound for the aroma formation of fragrant rice [5], and its content in rice grains is used to determine whether it is fragrant rice. Fragrant rice has been proven to contain aromatic substances 2-AP in its body, except for its roots. In the synthesis of 2-AP, it is believed that proline and ornithine are precursors of 2-AP [6]. The nitrogen source of 2-AP is proline, glutamic acid, and ornithine, and the basic carbon frames of proline and ornithine are derived from glutamic acid. While 1-pyrroline is the direct precursor of 2-AP, Δ1-pyrroline is formed from γ-Aminobutyraldehyde as the precursor [7]. In non-aromatic rice, γ-Aminobutyraldehyde is catalyzed to 4-aminobutyric acid (GABA) due to the presence of the *BADH2*. However, mutations in the *BADH2* in aromatic rice lead to the inability to synthesize *BADH2* normally, so γ-Aminobutyraldehyde continues accumulating, thereby contributing to the synthesis of 2-AP [8]. Figure 1 shows several potential synthesis pathways of aroma substance 2-AP. Figure 1 shows that under the action of enzymes, Orn is catalyzed to form putrescine, and putrescine reacts with 1-P to form 2-AP through self-cyclization under the catalysis of DAO. Glu and Pro are catalyzed by P5CS and PDH, respectively, to form P5C, which reacts with MG through a non-enzyme pathway to form 2-AP. Orn can also form P5C under the catalysis of OAT and participate in the synthesis pathway of 2-AP.

The aroma content in fragrant rice is not only influenced by gene regulation but also influenced by external agronomic practices. Luo et al. [9] improved the aroma content of fragrant rice by changing farmland irrigation conditions, leading to increased proline accumulation and enhanced expression of the aroma-related gene ProDH. Xie et al. [10] also sprayed GABA on the leaves, resulting in increased aroma of fragrant rice grains during the filling period and improved stress resistance ability of fragrant rice. Luo et al. [11] found that the application of the fungicide epoxiconazole significantly increased the aroma content and yield of fragrant rice, while reducing chalkiness of rice grains. Furthermore, it improved the protein and amylose content of fragrant rice grains, thereby enhancing its quality. Previous studies have shown that spraying paclobutrazol on the leaves increased the aroma content and yield of fragrant rice, as well as the net photosynthetic rate during the filling stage [12].

Plant growth regulators are essential in improving plant growth status, yield, and quality [13]. As a class of plant growth regulators, triazole compounds can enhance the tolerance of plants to various abiotic stresses by increasing the content of antioxidants in stressed plants [14]. As a triazole compound with plant growth regulation characteristics, paclobutrazol can affect the isoprenoid metabolism pathway in plants and change the state of plant hormones by inhibiting gibberellin synthesis, reducing ethylene content, and promoting cytokinin synthesis [15]. Some studies have shown that paclobutrazol can maintain the chlorophyll content and photosynthetic rate of soybeans under drought stress by increasing the range of auxin and zeatin in soybeans and improving the drought resistance ability of soybeans [16]. Applying paclobutrazol to rapeseed improved carbohydrate utilization rate, reduced plant height, and facilitated mechanical harvesting [17]. In addition to regulating hormone balance, paclobutrazol can increase various antioxidants in rice to cope with multiple abiotic stresses. For example, Pan et al. [18] found that foliar spraying of paclobutrazol increased the activities of peroxidase (POD) and superoxide dismutase (SOD) in rice and decreased the content of malondialdehyde (MDA). Paclobutrazol also expands the scope of proline in rice [12]. Proline helps plants regulate intracellular osmotic pressure and improve their tolerance to abiotic stresses, but it also is an essential precursor of aroma synthesis substance 2-AP in fragrant rice [8].

While previous studies have demonstrated that spraying paclobutrazol on the leaves during the filling stage significantly improves the net photosynthetic rate and aroma content of fragrant rice grains, there is no further explanation regarding how paclobutrazol enhances the aroma synthesis mechanism of fragrant rice [12]. This experiment was conducted to investigate the internal mechanism of effects of paclobutrazol on aroma synthesis of fragrant rice and its application methods in improving the quality of fragrant rice seedlings by initiating seeds with paclobutrazol, measuring aroma and its related physiological and biochemical indicators, as well as changes in the expression of aroma related genes.

## Results

### Growth attributes

**Fig. 2:**
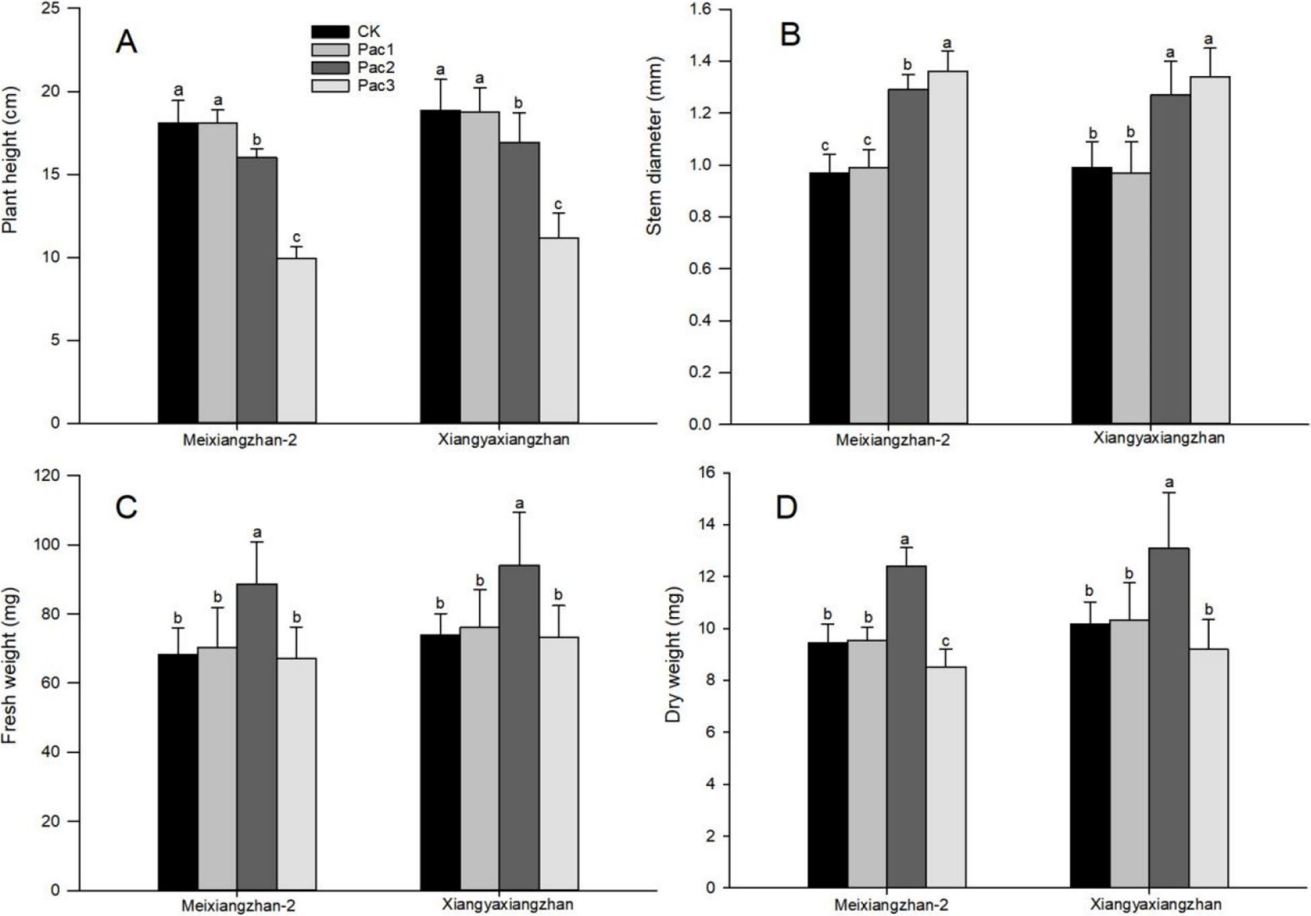
Effect of different paclobutrazol concentrations on growth attributes of fragrant rice seedlings [Plant height (**A**), stem width (**B**), fresh weight (**C**) and dry weight (**D**)] in fragrant rice seedlings. Each column represents the mean of three data ± standard errors (*n* = 3). Bars sharing a typical letter do not differ significantly at *p* < 0.05

After seed priming with paclobutrazol, the growth attributes of fragrant rice seedlings differed significantly among different treatments. Figure 2 shows that paclobutrazol treatment significantly reduced the plant height of fragrant rice seedlings and increased stem diameter. Compared with CK, the plant height of Meixiangzhan-2 and Xiangyaxiangzhan under the Pac3 decreased by 45.07% and 40.69%, respectively, and the stem diameter increased by 40.21% and 35.35%. Under the treatment of paclobutrazol, the fresh weight and dry weight of fragrant rice seedlings showed a trend of increasing first and then decreasing. Compared to CK, the fresh weight of Meixiangzhan-2 and Xiangyaxiangzhan under the Pac2 increased by 30.05% and 27.19%, and the dry weight increased by 31.35% 28.69%, respectively.

### Photosynthetic pigments and photosynthetic properties

**Fig. 3:**
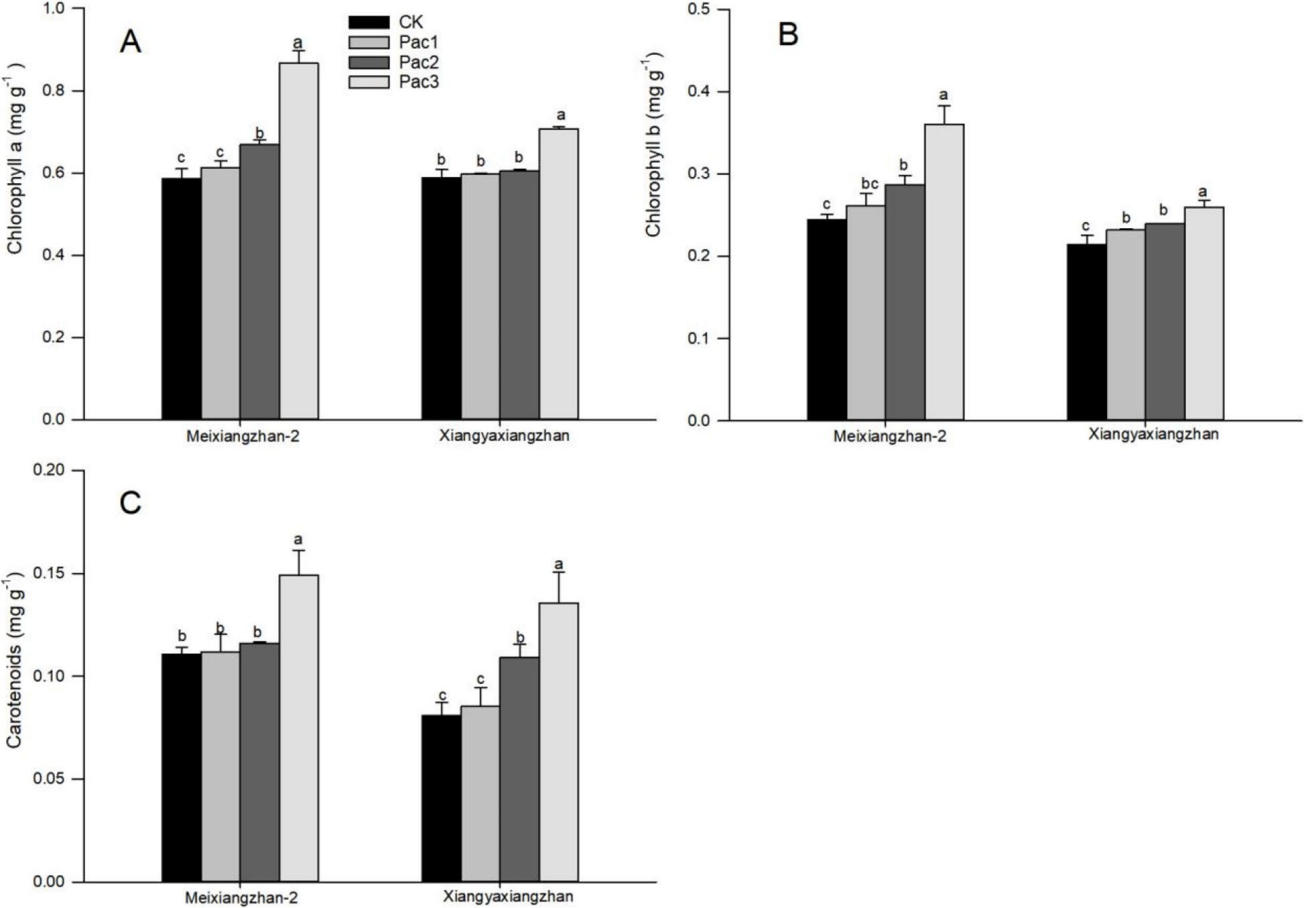
Effect of different paclobutrazol concentrations on photosynthetic pigments [Chlorophyll a (**A**), Chlorophyll b (**B**) and Carotenoids (**C**)] in fragrant rice seedlings. Each column represents the mean of three data ± standard errors (*n* = 3). Bars sharing a typical letter do not differ significantly at *p* < 0.05

The content of photosynthetic pigments in fragrant rice seedlings treated with paclobutrazol seed priming significantly increased. Figure 3 shows that chlorophyll a, b, and carotenoids in Meixiangzhan2 and Xiangyaxiangzhan under treatment increased significantly, with the highest content in Pac3, with chlorophyll increasing by 47.54% and 20.06%, chlorophyll b increasing by 47.52% and 21.40%, carotenoids increased by 34.89% and 67.18%, respectively, compared to CK.

**Fig. 4:**
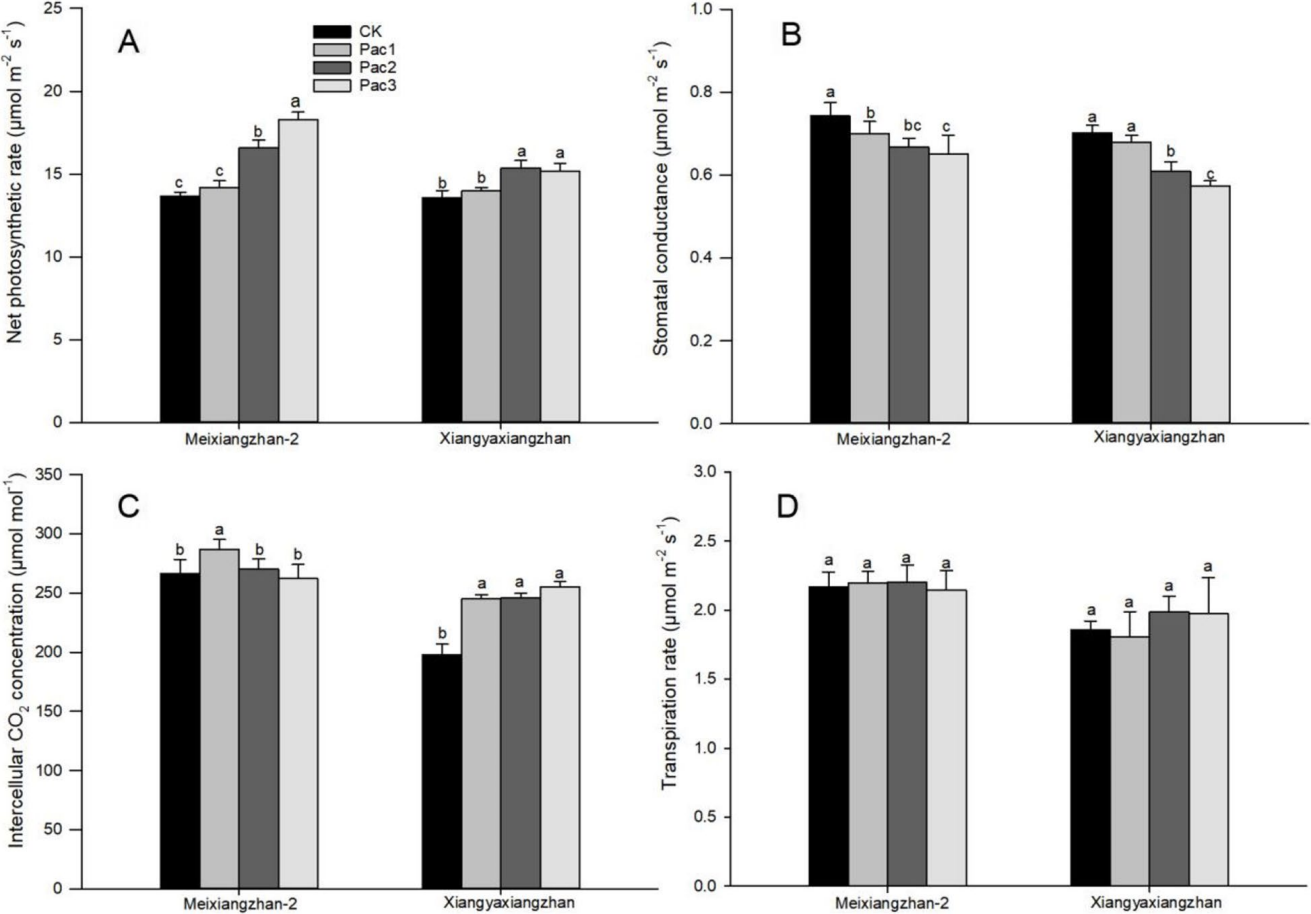
Effect of different paclobutrazol concentrations on photosynthetic properties [Net photosynthetic rate (**A**), Stomatal conductance (**B**), Intercellular CO_2_ concentration (**C**) and Transpiration rate (**D**)] in fragrant rice seedlings. Each column represents the mean of three data ± standard errors (*n* = 3). Bars sharing a typical letter do not differ significantly at *p* < 0.05

As can be seen from Fig. 4, paclobutrazol treatment significantly increased the net photosynthetic rate of fragrant rice seedlings but significantly decreased stomatal conductance. For Meixiangzhan-2, the change of intercellular CO_2_ concentration showed a trend of first increasing and then decreasing with the increase of paclobutrazol concentration. In contrast, for Xiangyaxiangzhan, it increased with the increase in concentration. The transpiration rate of fragrant rice seedlings was affected by paclobutrazol, and there were differences among treatments, but they did not reach a significant level. The net photosynthetic rate of Pac3 of Meixiangzhan-2 increased by 33.68% compared to CK, while the stomatal conductance decreased by 12.35%. The net photosynthetic rate of Pac2 of Xiangyaxiangzhan was the highest, increased by 13.05% compared to CK, and the stomatal conductance of Pac3 was the lowest, decreased by 18.14%. Among the intercellular CO_2_ concentrations, Pac1 of Meixiangzhan-2 was the highest, increased by 7.84% compared to CK, and Xiangyaxiangzhan Pac2 was the highest, increased by 29.29%.

### 2-AP Content

**Fig. 5:**
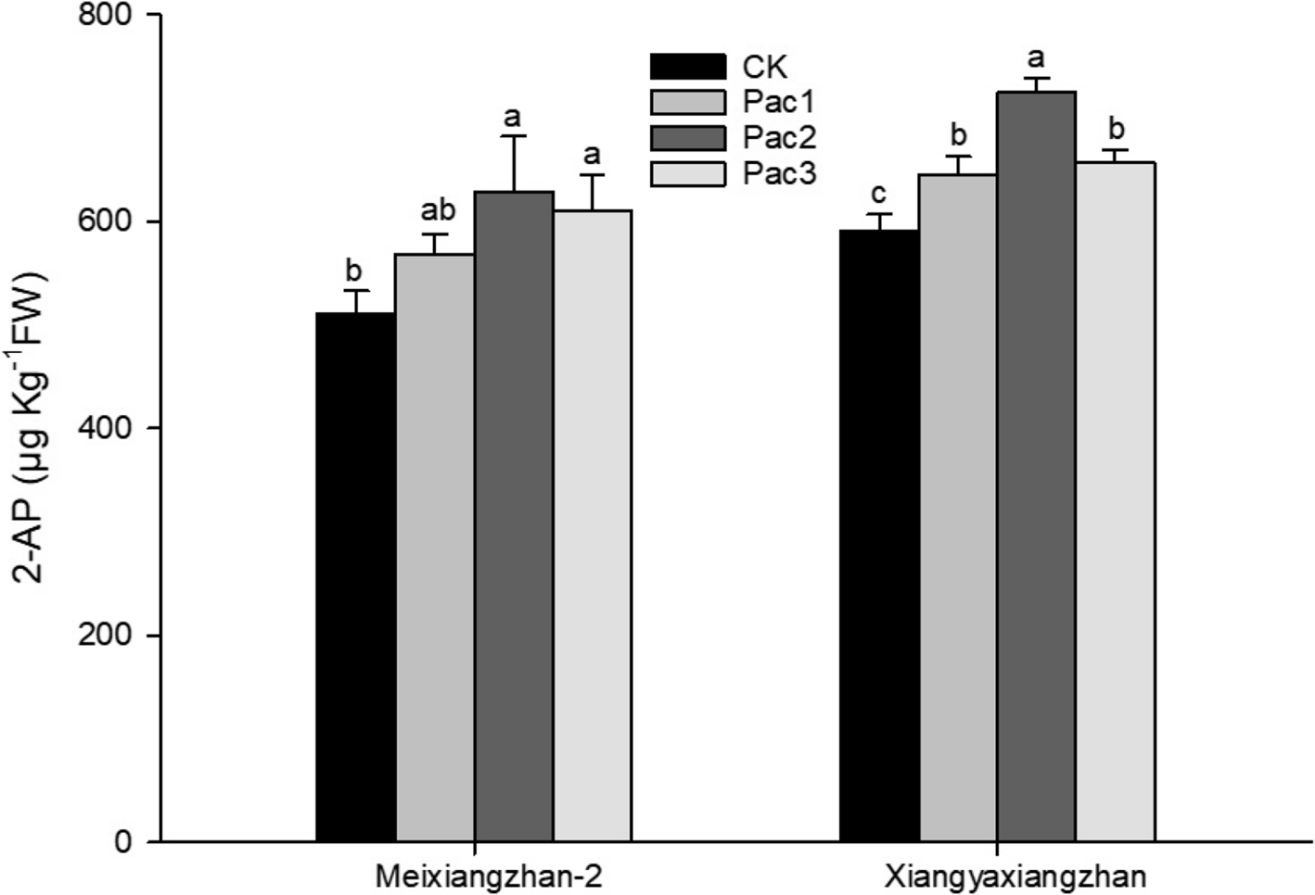
Effect of different paclobutrazol concentrations on 2-AP. Each column represents the mean of three data ± standard errors (*n* = 3). Bars sharing a typical letter do not differ significantly at *p* < 0.05

Paclobutrazol treatment significantly increased the aroma substance 2-AP in fragrant rice seedlings. As shown in Fig. 5, the 2-AP content of Meixiangzhan-2 and Xiangyaxiangzhan showed a trend of increasing and then decreasing with the increase of paclobutrazol concentration. Both varieties had the highest 2-AP content in Pac2, which increased by 22.91% and 22.65%, respectively, compared to CK.

### Aroma-related precursors and aroma enzymes

**Fig. 6:**
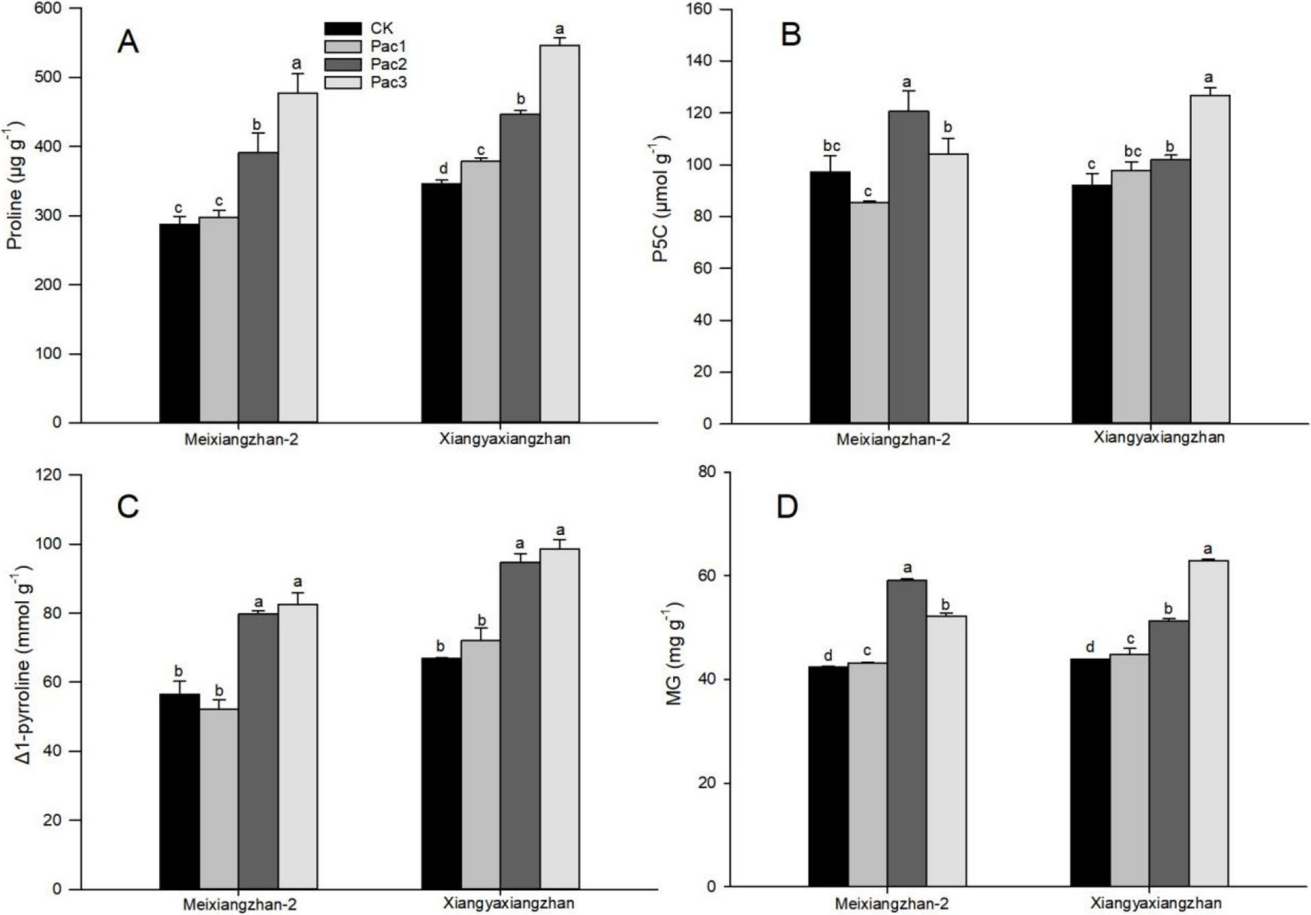
Effect of different paclobutrazol concentrations on aroma-related precursors [Proline (**A**), P5C (**B**), Δ1-pyrroline (**C**) and MG (**D**)] in fragrant rice seedlings. Each column represents the mean of three data ± standard errors (*n* = 3). Bars sharing a typical letter do not differ significantly at *p* < 0.05

The effect of seed priming with paclobutrazol on the aroma-related precursors of fragrant rice seedlings was significant. As shown in Fig. 6, the treatment significantly increased the contents of proline and Δ1-pyrroline in Meixiangzhan-2 and Xiangyaxiangzhan. The change of P5C content in Meixiangzhan-2 presents a trend of first decreasing, then increasing and then decreasing with the increase of paclobutrazol concentration. In contrast, the change of MG presents a trend of first increasing, then decreasing. The changes in P5C and MG content inXiangyaxiangzhan increased with the increase in concentration. Among the proline and Δ1-pyrroline indicators, both Meixiangzahn-2 and Xiangyaxiangzhan had the best content in Pac3. Compared to CK, proline increased by 66.46% and 58.01%, and Δ1-pyrroline increased by 46.17% and 47.39%, respectively, in the two varieties. The P5C and MG content of Meixiangzhan-2 were both the highest in Pac2, increased by 24.16% and 39.12% compared to CK, and in Xiangyaxiangzhan, Pac3 had the highest, increased by 37.83% and 43.39%.

**Fig. 7:**
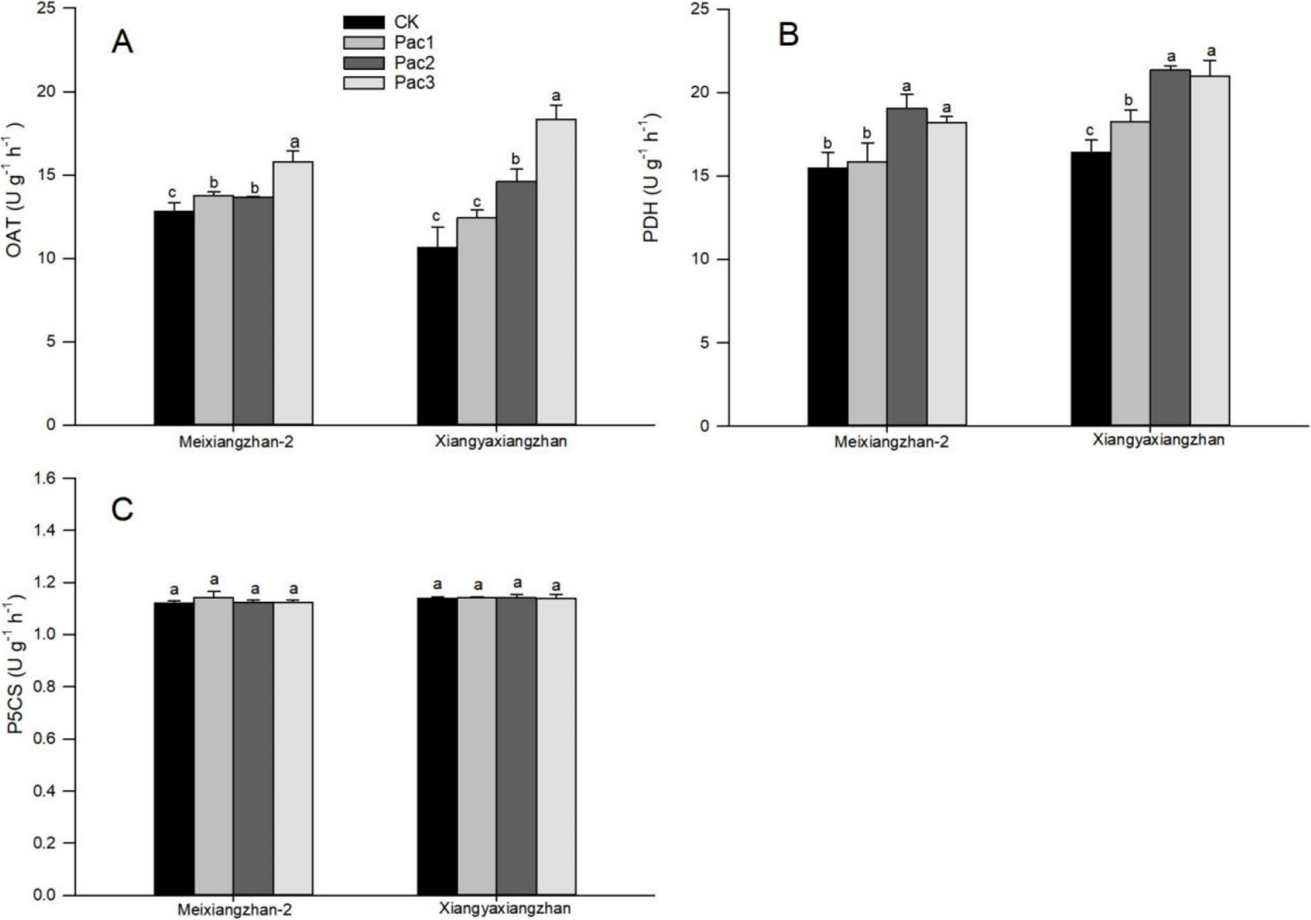
Effect of different paclobutrazol concentrations on aroma enzymes [OAT (**A**), PDH (**B**) and P5CS (**C**)] in fragrant rice seedlings. Each column represents the mean of three data ± standard errors (*n* = 3). Bars sharing a typical letter do not differ significantly at *p* < 0.05

The effects of seed priming with paclobutrazol on the activities of three aroma enzymes in fragrant rice seedlings were different. Figure 7 shows that paclobutrazol significantly increased the activity of OAT, with the highest enzyme activity in Pac3 of Meixiangzhan-2 and Xiangyaxiangzhan, which increased by 23.28% and 72.05%, respectively, compared to CK. The effect of paclobutrazol on the PDH of fragrant rice seedlings showed an increasing and decreasing trend. Compared to CK, Pac2, which had the highest enzyme activity in the two varieties, increased by 23.23% and 30.18%, respectively, but paclobutrazol had no significant impact on the enzyme activity of P5CS.

### Osmoregulatory substances and resistant enzymes

**Fig. 8:**
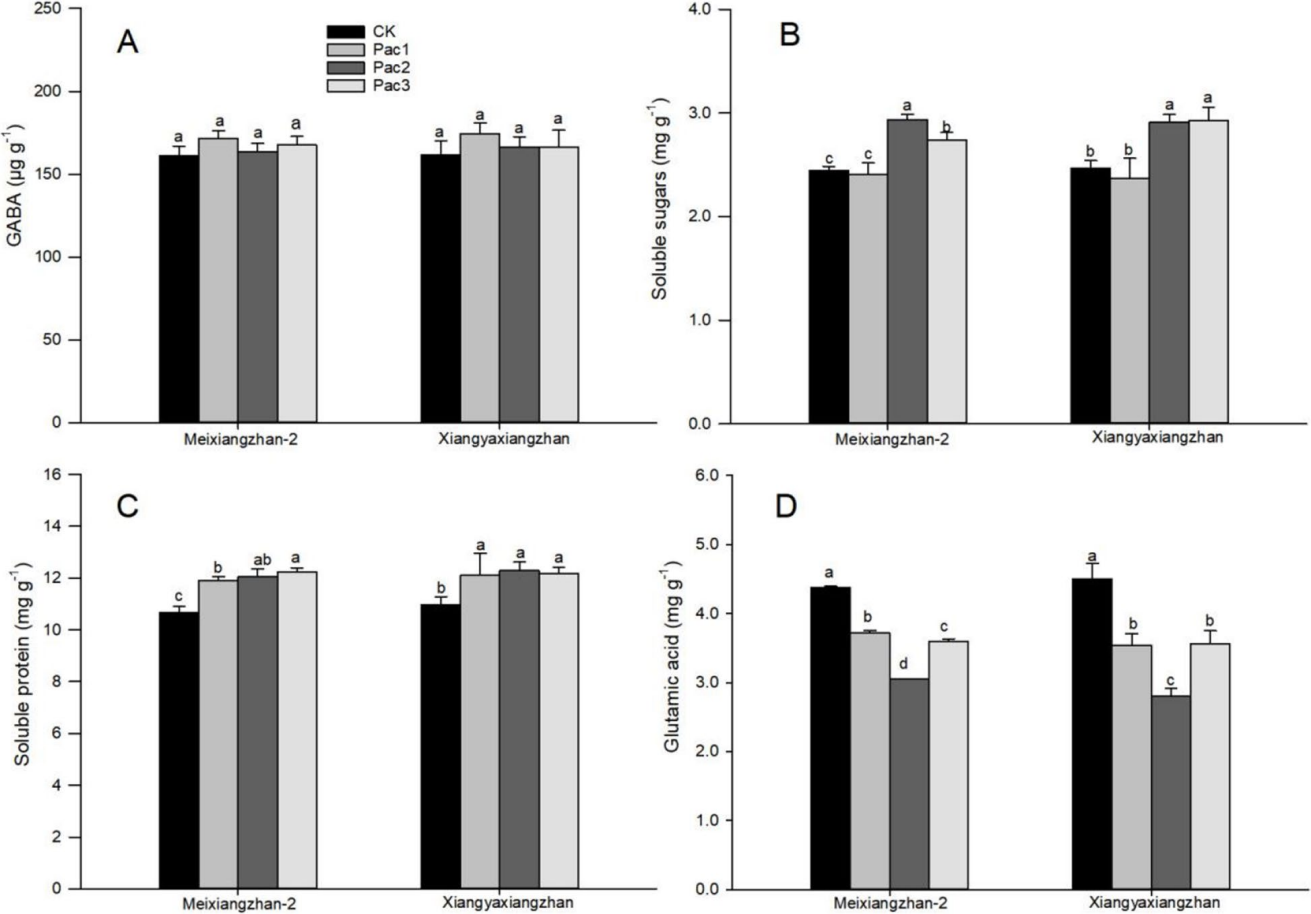
Effect of different paclobutrazol concentrations on osmoregulatory substances [GABA (**A**), Soluble sugars (**B**) Soluble protein (**C**) and Glutamic acid (**D**)] in fragrant rice seedlings. Each column represents the mean of three data ± standard errors (*n* = 3). Bars sharing a typical letter do not differ significantly at *p* < 0.05

The effect of seed priming with paclobutrazol on osmoregulation substances in fragrant rice seedlings is shown in Fig. 8. The effect of paclobutrazol on GABA is insignificant, but both increase the soluble protein and sugar content. The effect of paclobutrazol on the glutamic acid content in both varieties decreases first and then increases as the concentration increases. The highest soluble sugar content of Meixiangzhan-2 was in Pac2, and the highest soluble protein content was in Pac3, which increased by 20.00% and 14.82%, respectively, compared to CK. The highest soluble sugar content of Xiangyaxiangzhan was in Pac3, and the highest soluble protein content was in Pac2, which increased by 18.80% and 11.95%, respectively, compared to CK. Among the glutamic acid, the lowest content of both varieties was Pac2, which decreased by 30.16% and 37.78%, respectively, compared to CK.

The effect of paclobutrazol on resistant enzymes in fragrant rice seedlings is shown in Fig. 8. It significantly reduces the content of MDA in the seedlings and increases the enzyme activities of resistant enzymes SOD, POD, and CAT. Compared with CK, Meixiangzhan-2 has a minimum decrease of 56.79% in MDA and a maximum increase of 39.27%, 18.83%, and 41.82% in SOD, POD, and CAT enzyme activities, respectively. Xiangyaxiangzhan has a minimum decrease of 54.74% in MDA and a maximum increase of 45.47%, 37.49%, and 34.23% in SOD, POD, and CAT enzyme activities, respectively.

### Expression levels of genes related to 2-AP biosynthesis

**Fig. 9:**
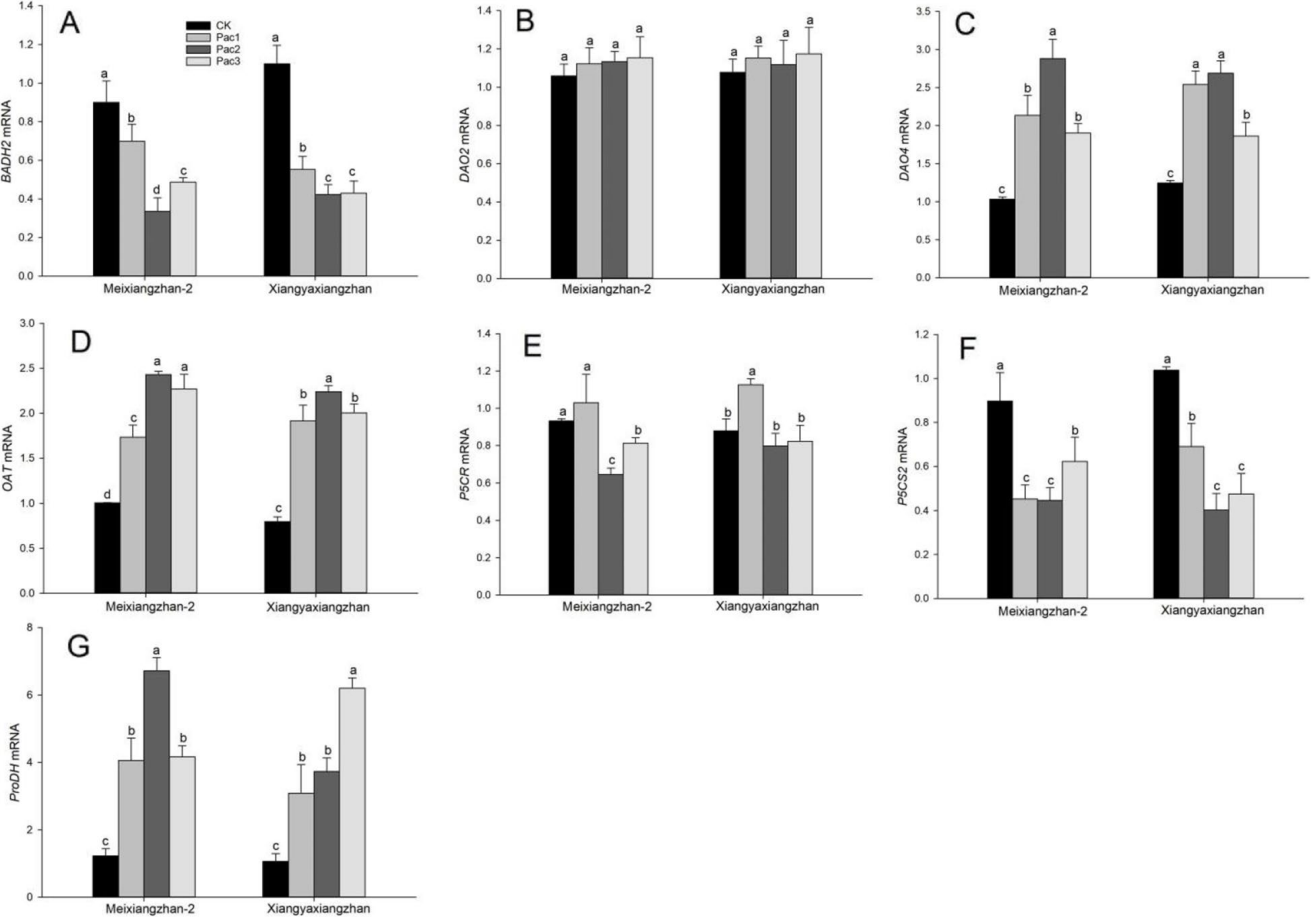
Effect of different paclobutrazol concentrations on resistant enzymes [MDA (**A**), SOD (**B**), POD (**C**) and CAT (**D**)] in fragrant rice seedlings. Each column represents the mean of three data ± standard errors (*n* = 3). Bars sharing a typical letter do not differ significantly at *p* < 0.05

Figure 9 shows the expression level of aroma-related genes in fragrant rice seedlings treated with paclobutrazol seed priming. The expression levels of *BADH2* and *P5CS2* were significantly downregulated under the influence of paclobutrazol. Compared to CK, Meixiangzhan-2 decreased by 22.32 ~ 62.64% and 30.68 ~ 50.29%, respectively, and Xiangyaxiangzhan decreased by 16.39 ~ 64.12% and 27.95 ~ 57.04%. The expression levels of *DAO4*, *OAT*, and *ProDH* were significantly increased under the influence of paclobutrazol, with Meixiangzhan-2 increased by 84.24 ~ 178.94%, 72.47 ~ 141.92%, and 231.71 ~ 447.76%, respectively, and Xiangyaxiangzhan increased by 79.79 ~ 178.69%, 71.62 ~ 144.54%, and 227.46 ~ 439.78%. The expression of *P5CR* in Meixiangzhan-2 showed an increasing and decreasing trend, but there was no significant difference between Pac1 with increased expression and CK. Xiangyaxiangzhan also showed a trend of first increasing and then decreasing, but there was no statistical difference between Pac2 and 3 with decreased expression and CK. Meixiangzhan-2 Pac2 had the lowest expression of *P5CR*, decreased by 30.59% compared to CK, and Xiangyaxiangzhan Pac1 had the highest expression, increased by 16.82. The effect of paclobutrazol on the expression of *DAO2* was not significant between the two varieties.

### Correlation analysis and principal component analysis

**Fig. 10:**
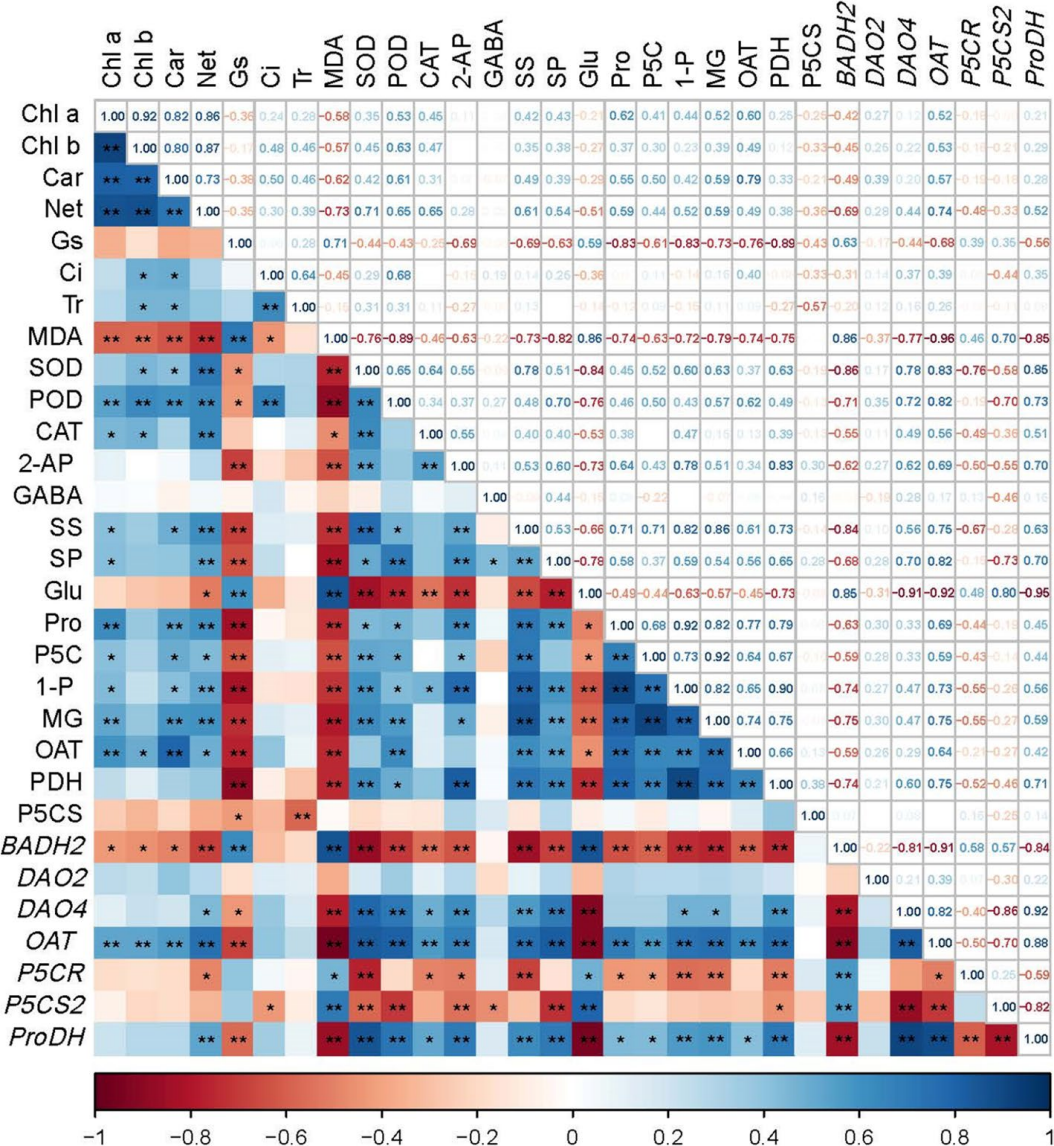
The heatmap for the investigated parameters. “*” show *p* < 0.05, and “**” show *p* < 0.01. The number represents the r value. Chl a, Chlorophyll a; Chl b, Chlorophyll b; Car, Carotenoids; Net, Net photosynthetic rate; Gs, Stomatal conductance; Ci, Intercellular CO_2_ concentration; Tr, Transpiration rate; SS, Soluble sugars; SP, Soluble protein; Glu, glutamic acid contents; Pro, Proline; 1-P, Δ1-pyrroline contents

The correlation heat map between photosynthetic pigments, photosynthetic characteristics, aroma-related enzymes, precursors, osmoregulation substances, resistant enzymes, and aroma-related gene expression levels and aroma substances 2-AP is shown in Fig. 10. The content of 2-AP was negatively correlated with MDA, positively correlated with the activity of resistant enzymes, negatively correlated with glutamate content, correlated with Δ1-pyrroline, negatively correlated with the expression of *BADH2*, *P5CR*, and *P5CS2*, and positively correlated with the expression of *DOA4*, *OAT*, and *ProDH*. Proline content was positively correlated with aroma, photosynthetic pigments, net photosynthetic rate, soluble sugar, and protein, negatively correlated with glutamic acid, and positively correlated with aroma precursors and aroma enzymes.

**Fig. 11:**
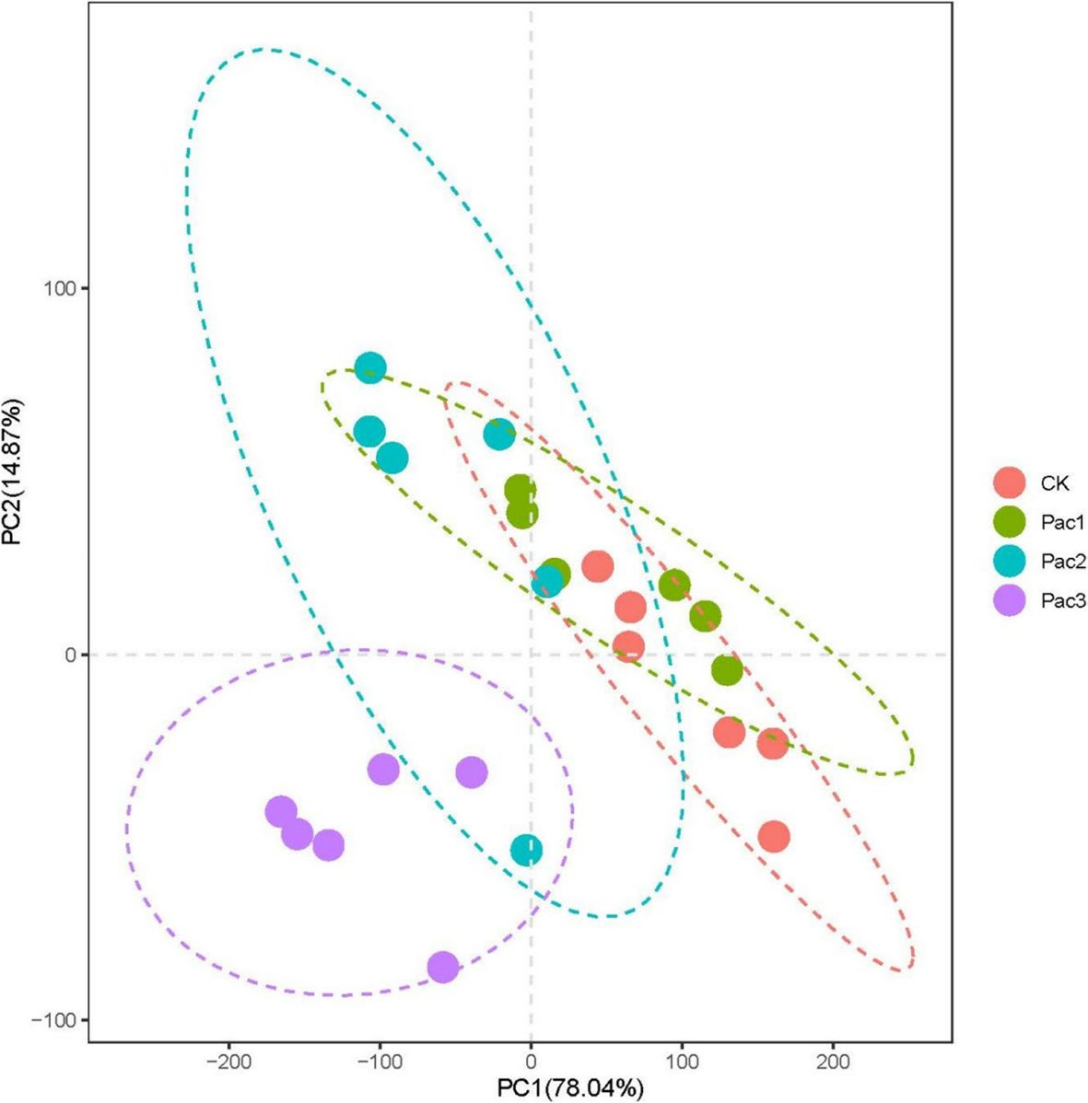
The principal component analysis plot for the investigated parameters

The principal component analysis of photosynthetic pigments, photosynthetic characteristics, aroma-related enzymes and precursors, osmoregulation substances, resistant enzymes, aroma-related gene expression, and aroma substance 2-AP is shown in Fig. 11. The cumulative contribution rate of the first and second principal components reached 92.91%. PC1 is mainly contributed by proline (-0.8275), 2-AP (-0.5262), and net photosynthetic rate (-0.8507). PC2 is mainly associated with *P5CS2* (0.9945), glutamic acid (0.4.385), and P5C (-0.6633). Pac3 scored significantly lower on the first and second principal components than CK because of its higher aroma and proline content, net photosynthetic rate, and lower glutamic acid content and *P5CS2* expression.

## Discussion

**Fig. 12:**
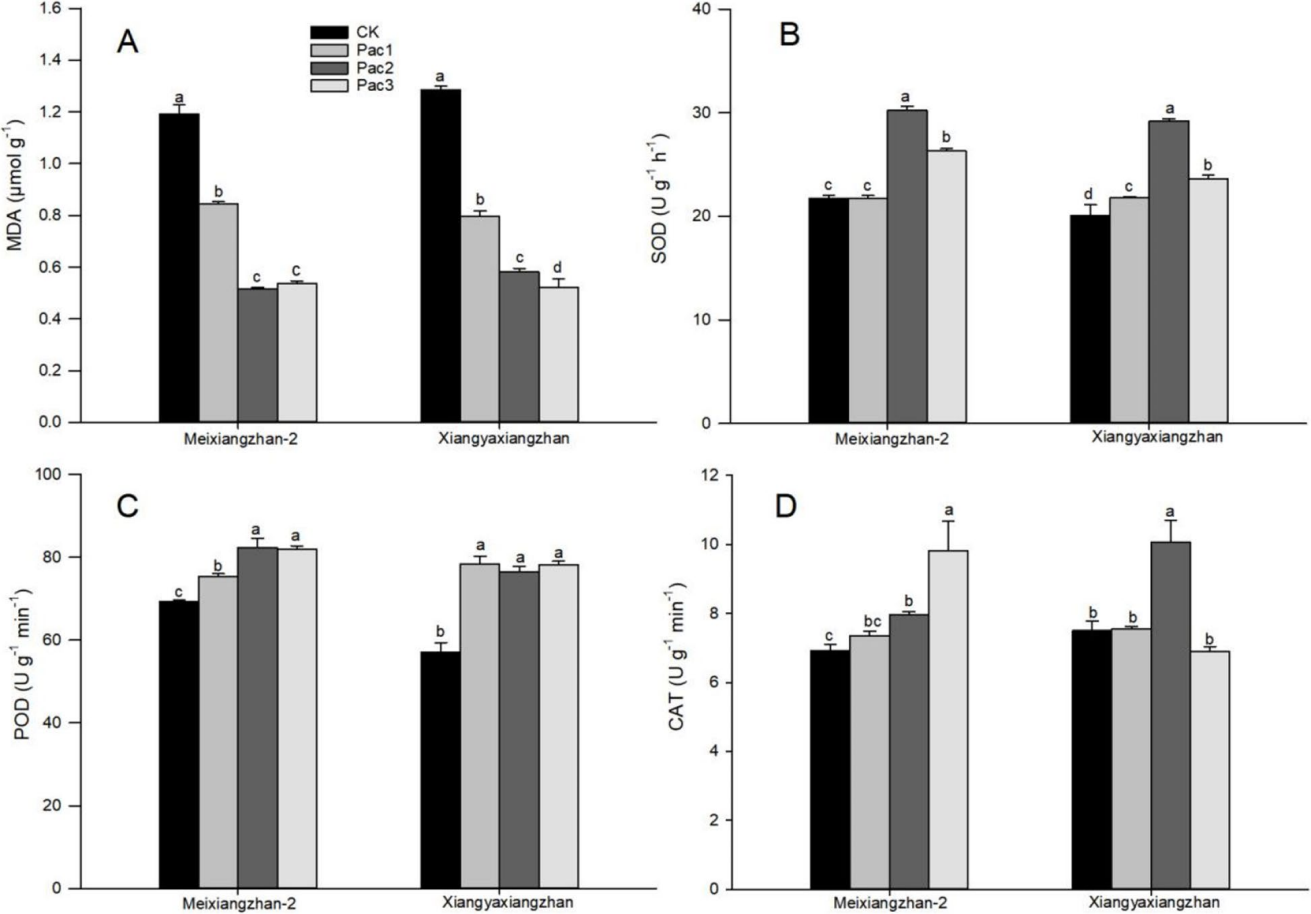
Effects of different trans-zeatin concentrations on expression levels of genes related to 2-AP biosynthesis [*BADH2* (**A**), *DAO2* (**B**), *DAO4* (**C**), *OAT* (**D**), *P5CR* (**E**), *P5CS2* (**F**), and *ProDH* (**G**)] in fragrant rice seedling. Each column represents the mean of three data ± standard errors (*n* = 9). Bars sharing a typical letter do not differ significantly at *p* < 0.05

Previous studies [19] have confirmed that the application of paclobutrazol in plants can maintain the structural integrity of chloroplasts under drought conditions by enhancing the activity of antioxidant enzymes and limiting lipid peroxidation, and increasing the content of photosynthetic pigments such as chlorophyll a, chlorophyll b, and carotenoids. This study also confirmed that the content of photosynthetic pigments and the net photosynthetic rate of seedlings increased under the treatment of seed initiation with paclobutrazol. This result is consistent with previous field experiments [12] in which spraying paclobutrazol during the filling stage increased the photosynthetic pigment content and net photosynthetic rate of aromatic rice leaves. The experimental results show that (Fig. 4) paclobutrazol significantly improves the net photosynthetic rate of seedlings by reducing leaf stomatal conductance and increasing intercellular carbon dioxide concentration. According to research, paclobutrazol inhibits the synthesis of gibberellins in plants by deactivating cytochrome P450-dependent monooxygenases and preventing the oxidation of kaurene to kaurenoic acid. When gibberellin synthesis is disrupted, more precursors accumulate and divert in the terpenoid pathway, leading to the production of abscisic acid. By affecting the balance of hormones in plants, paclobutrazol plays a role in regulating plant growth [20]. As a result, the treatment of paclobutrazol significantly reduces the plant height of seedlings (Fig. 2) and increases the stem diameter, fresh weight, and dry weight of the seedlings. Therefore, an appropriate concentration of PBZ can improve the growth attributes of rice seedlings. Further experimental evidence is needed to confirm the specific changes in hormones within the seedlings. At the same time, the activity of resistant enzymes in seedlings was increased, and the content of MDA was reduced (Fig. 12), consistent with the experimental results of Pan et al. [18]. Previous studies [21] have shown that increasing the content of photosynthetic pigments and the activity of resistant enzymes (SOD, POD, and CAT) in rice seedlings is beneficial to improving the stress resistance of the seedlings. The results of this experiment confirmed that seed initiation with paclobutrazol can enhance the ability of fragrant rice seedlings to resist adverse.

Various methods can be used to increase the accumulation of aroma-critical volatile compound 2-AP in fragrant rice [22–24]. This experiment confirmed that seed priming with paclobutrazol can increase the content of 2-AP in fragrant rice seedlings. At the same time, the contents of proline, P5C, 1-pyrroline, and MG, the aroma precursors, were significantly increased. This result further confirmed that proline is an essential precursor for aroma synthesis and has a significant positive correlation with 2-AP and aroma prerequisite substances, consistent with previous research results [9]. However, there is a negative correlation between proline content and 2-AP content, and glutamic acid. Although glutamic acid and proline are precursors of 2-AP, proline plays a more critical role in aroma synthesis [25]. Similar to the experimental results of spraying trans zeatin on fragrant rice seedlings, the glutamic acid content in fragrant rice seedlings decreased [26]. However, the content of proline and 2-AP significantly increased. This result may indicate an antagonistic effect between proline and glutamic acid in plants, which is also consistent with the conclusion of Woodrow et al. [27]. Moreover, the expression of aroma-related genes showed a significant decrease in *P5CS2* expression, similar to the change in *BADH2* gene expression, and a significant negative correlation with the change in 2-AP. Although Fig. 7 shows no significant change in the enzyme activity of P5CS regulated by *P5CS2*, it can promote the mutual conversion of P5C and glutamic acid. We speculate that the decrease in glutamic acid content reduces the conversion of glutamic acid to P5C, downregulating the expression of *P5CS2*, which controls the synthesis of the invertase P5CS. The 2-AP synthesis pathway tends to be the proline pathway, which promotes the upregulation of the expression of *DAO4*, *OAT*, and *ProDH*. It increases the enzyme activity of OAT and PDH, thereby increasing the content of 2-AP.

Proline is an essential precursor of aroma synthesis and participates in plant stress resistance physiology, increasing plant stress resistance [28, 29]. The treatment of paclobutrazol not only increased the seedling quality of fragrant rice and improved the enzyme activity of resistant enzymes but also increased the content of proline, a stress-resistant substance, and increased the content of soluble sugar and soluble protein, an osmotic regulator. Our research further confirms the use value of paclobutrazol in the agricultural field. Moreover, more experiments can be conducted to study the impact of paclobutrazol on other rice growth stages. The half-life of tebuconazole in soil is over six months. Studies have shown [30] that the uncontrolled use of triazole plant growth regulators leads to varying degrees of residual regulators in soil and water sources. The residual tebuconazole can also have negative effects on plants and pose potential hazards to the environment and human health. The application of tebuconazole through seed treatment can prevent it from directly entering the natural environment and reduce the residual tebuconazole in the environment. However, compared with direct spraying of tebuconazole, the impact of tebuconazole seed treatment on the environment needs further research.

## Conclusion

The application of the paclobutrazol resulted in improved quality of aromatic rice seedlings and increased levels of 2-AP. Furthermore, the treatment exhibited enhanced photosynthetic characteristics and increased stress tolerance in the rice seedlings. Additionally, there was an elevation in the concentration of proline, a key precursor involved in aroma synthesis. In summary, the recommended concentration for seed initiation of paclobutrazol is 40 mg·L^−1^ ~ 80 mg·L^−1^.

## Materials and methods

### Plant materials, growth condition, and experimental design

This experiment involved materials that were selected from fragrant rice varieties widely grown in South China: Meixiangzhan-2 and Xiangyaxiangzhan. The plant material used for the experiments was provided by the Rice Cultivation Research Office, College of Agriculture, South China Agricultural University. Before planting, rice seeds were soaked in a particular concentration of paclobutrazol solution. The concentration of the paclobutrazol solution was divided into three concentration gradients: (1) Pac 1(20 mg·L^−1^); (2) Pac 2(40 mg·L^−1^); (3) Pac 3(80 mg·L^−1^). Seeds were primed with water as a control (CK) after being soaked in the above solution at room temperature for 24 h. They were taken out, washed with clean water, dried at room temperature for 24 h, and subjected to seed germination treatment. After the seeds germinated, they were transferred into pots containing 10 kg of rice soil and placed in an artificial climate box (25 ℃, 60% relative humidity, and 100 μmol m^−2^ s^−1^ for 16 h and dark for 8 h) at the College of Agriculture, South China Agricultural University.

After growing under the above conditions for 15 days, healthy and similar fragrant rice seedlings were selected from each treatment. Samples were stored at a low temperature of -80℃ for subsequent physiological, biochemical, and molecular testing. Fifty fragrant rice seedlings were selected from each treatment to test the quality of rice seedlings, including stem diameter, plant height, fresh weight, and dry weight.

### Determination of photosynthetic pigments and photosynthetic properties

The measured photosynthetic pigments included chlorophyll a, chlorophyll b, and carotenoids. The determination method was referred to Luo et al. [31]. The sample to be tested was taken and placed in a centrifuge tube, and the acetone ethanol mixture was added. It was then stored in the dark for 24 h. After that, an ultraviolet spectrophotometer was used to compare the color at wavelengths of 665, 649, and 470 nm, using the extract as a blank control.

The measured photosynthetic properties included net photosynthetic rate, stomatal conductance, intercellular CO_2_ concentration, and transpiration rate. The determination method was referred to Luo et al. [11]. Before sampling, a portable photosynthesis meter (LI-6800, LI-COR, USA) was used to measure the fragrant rice seedlings to be tested at 9:00–11:00 a.m.

### Determination of 2-AP, aroma-related precursors and aroma enzymes

The determination of 2-AP was based on the method of Mo et al. [32]. Dichloromethane was used for ultrasonic extraction of the sample to be tested, and GCMS-QP 2010 Plus (Shimadzu Corporation, Japan) was used to determine the extracted sample. The chromatographic column model was RESTEK Rxi-5 m (30 m × 0.32 mm × 0.25 μm). The unit of 2-AP content was μg·g^−1^.

The aroma-related substances measured included proline, P5C, Δ1-pyrroline, and MG. The determination of proline and P5C was based on the method of Li et al. [33]. The sample to be tested was put into a centrifuge tube, and glacial acetic acid and acidic ninhydrin were added in turn for a color reaction. A spectrophotometer was used to determine the absorbance value at a wavelength of 520 nm to obtain the proline content, with the unit of μg·g^−1^. Sulfosalicylic acid and 2-amino benzaldehyde were used to react with the sample, which was then centrifuged to take the supernatant. A spectrophotometer was used to determine the absorbance value at a wavelength of 440 nm to obtain the P5C content, with the unit of μmol·g^−1^. Δ1-pyrroline was determined according to the method of Bao et al. [34]. The plant sample to be tested was added to 2-amino benzaldehyde for reaction. After the response, a spectrophotometer was used to measure the absorbance value at a wavelength of 430 nm to obtain the Δ1-pyrroline content in mmol·g^−1^. For the MG determination method, refer to Yadav et al. [35], the sample to be tested was reacted with dichlorobenzene and perchloric acid and then the absorbance value was measured at a wavelength of 336 nm to obtain the MG content, unit: μmol·g^−1^.

The aroma enzymes measured included PDH, OAT, and P5CS. Refer to Umair et al. [36] for the OAT determination method. Ornithine, pyridoxal phosphate, and after the reaction with α-ketoglutaric acid, 2-amino benzaldehyde was added for color development. The supernatant was taken for colorimetry, and the absorbance value was measured at a wavelength of 440 nm. The OAT activity was obtained with the enzyme activity unit of U·g^−1^·min^−1^. The determination methods of PDH and P5CS refer to Bao et al. [34]. The sample to be tested was reacted with proline cytochrome c, etc. After completion, 2-amino benzaldehyde was added for color development. The supernatant was taken for colorimetry, and the absorbance value was measured at a wavelength of 440 nm. The PDH activity was obtained, and the enzyme activity unit was U·g^−1^·min^−1^. The sample to be tested was reacted with ATP, glutamic acid, etc. After the reaction, the supernatant was taken for colorimetry, and the absorbance value was measured at a wavelength of 535 nm to obtain P5CS activity. The enzyme activity unit was U·g^−1^·min^−1^.

### Determination of osmoregulatory substances and resistant enzymes

The measured osmoregulation substances included GABA, soluble protein, soluble sugar, and glutamic acid. The determination method of GABA was based on the technique of Mo et al. [25]. The sample to be tested reacted with lanthanum chloride and sodium tetraborate. After the reaction, ethanol was added for color development. The absorbance value was measured at 645 nm to obtain the GABA content, with the unit of μg·g^−1^. The determination of soluble protein referred to the method of Ullah et al. [37]. Coomassie Brilliant Blue G-520 was added to the sample to be tested, fully developing the color, and then the absorbance value was measured at a wavelength of 595 nm. The soluble protein content was obtained through the standard curve, with the unit of mg·g^−1^. The soluble sugar content of the sample was determined by using the anthrone method for color development. After the color development, the soluble sugar content was measured at a wavelength of 620 nm, with the unit of mg·g^−1^. The sample to be tested was reacted with ninhydrin, the absorbance value was measured at a wavelength of 569 nm, and the Glu content was obtained through the standard curve, with the unit of mg·g^−1^.

The determination of resistant enzymes included MDA, SOD, POD, and CAT. The method referred to Li et al. [38]. The sample to be tested was reacted with thiobarbituric acid. After the reaction, the supernatant was taken and the absorbance value was measured at 532 nm, 600 nm, and 450 nm wavelengths, respectively. The MDA content was obtained in μmol·g^−1^. The sample to be tested was mixed with nitro tetrazolium nitride blue, methionine, and riboflavin, and reacted under 4000 lx sunlight. After the reaction was terminated, the absorbance was determined by colorimetry at a wavelength of 560 nm to obtain the SOD activity with the unit of U·g^−1^·h^−1^. The sample to be tested was quickly mixed with hydrogen peroxide and guaiacol solution, and then color comparison was performed at a wavelength of 470 nm. The data recording was started and the absorbance value was recorded every 30 s for 2 min to obtain the POD activity, with the unit of U·g^−1^·min^−1^. The sample to be tested was reacted with hydrogen peroxide, then a colorimetric test was performed at a wavelength of 240 nm. Data recording was started and the absorbance value was recorded every 30 s for 2 min to obtain the CAT activity, with the unit of U·g^−1^·min^−1^.

### Real-Time quantitative RT-PCR

The RNA analysis of the plant sample to be tested was performed using Luo et al. [9]. Total RNA was extracted using the HiPure Plant RNA Mini Kit (Magen, Guangzhou, China) and its quality and quantity were assessed by NanoDrop 2000. The Hiscript II QRT SuperMix for qPCR (+ gDNAwiper; Vazyme, Nanjing, China) synthesized cDNA from total RNA. Real-time quantitative RT-PCR (qRT-PCR) was conducted in the CFX96 real-time PCR System (Bio-Rad, Hercules, CA, United States). OsActin was used as an internal reference gene. Each RNA sample was performed in triplicate and a negative control without a cDNA template was always included. The primers used for qRT-PCR were designed using the software tool Primer 5 (Premier Biosoft International, Palo Alto, CA).

### Data analysis

In this study, R 4.1.2 (R Foundation for Statistical Computing, Vienna, Austria.) was used to perform a one-way analysis of variance (ANOVA) on the obtained data, correlation analysis, principal component analysis (PCA), and mapping. At a 5% probability level, the least significant (LSD) test was used to separate the differences between the averages. Other images were drawn using Sigma Plot 13.0 (Systat Software Inc., San Jose, CA, United States).

### Supplementary Information

**Additional file 1.**

## Data Availability

The original contributions presented in the study are included in the article, and further inquiries can be directed to the corresponding author.

## Abbreviations

2-AP: 2-Acetyl-1-pyrroline
P5CS: 1-Pyrroline-5-carboxylate synthetase
GABA: γ-Aminobutyric acid
PDH: Proline dehydrogenase
P5C: Pyrroline-5-carboxylic acid
BADH: Betaine aldehyde dehydrogenase
OAT: Ornithine transaminase
DAO: Diamine oxidase
SOD: Superoxide dismutase
POD: Peroxidase
CAT: Catalase
