# Supplementary material for: Effects of paclobutrazol seed priming on seedling quality, photosynthesis, and physiological characteristics of fragrant rice
Source: BMC Plant Biol. 2024 Jan 17;24:53. doi: 10.1186/s12870-023-04683-0 (PMC10792894; doi:10.1186/s12870-023-04683-0)
Supplement: Supplementary file 1 — Additional file 1. [file 12870_2023_4683_MOESM1_ESM.docx]

**Supplements**

1 The extracted RNA's quality


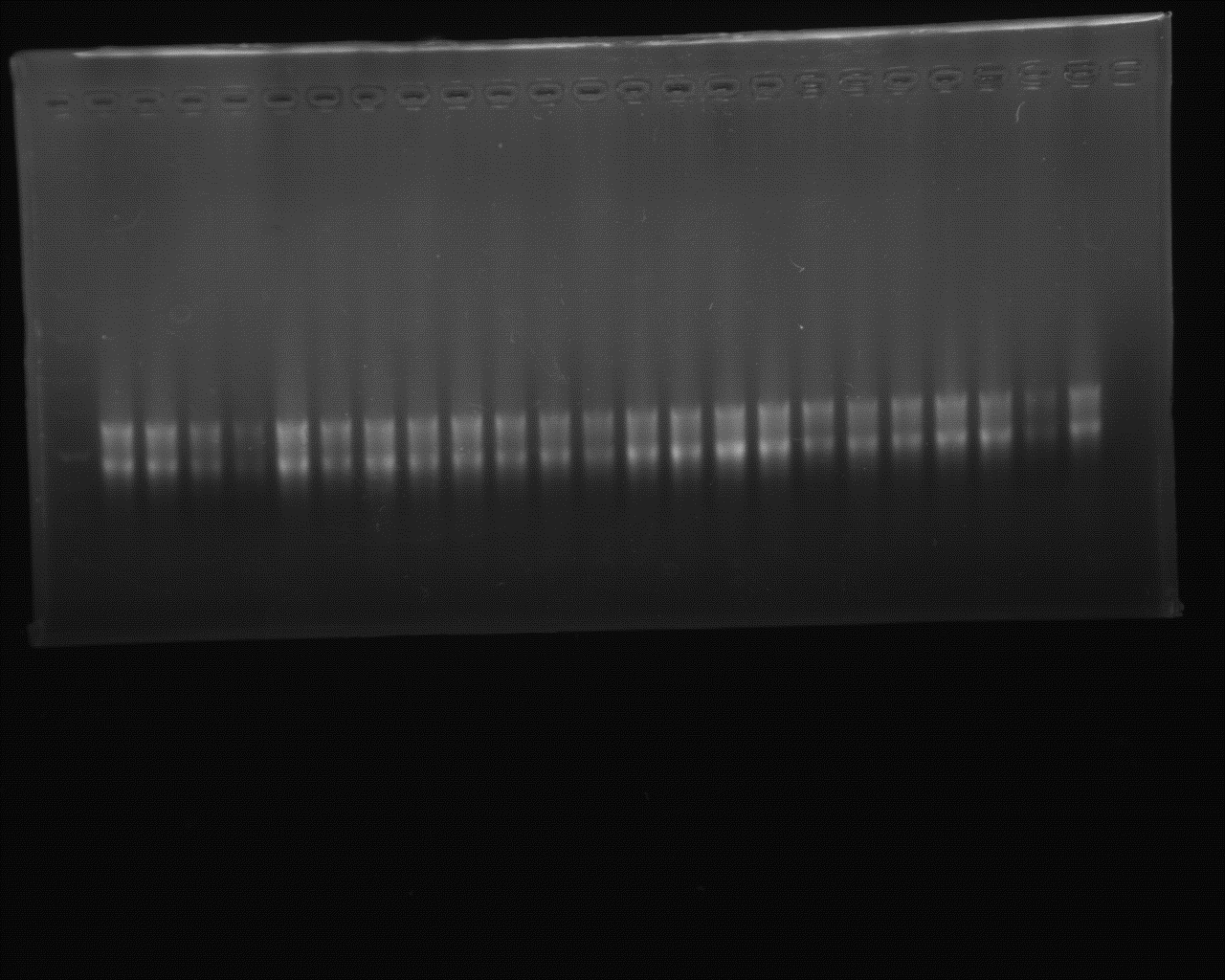


2 The qRT-PCR program details


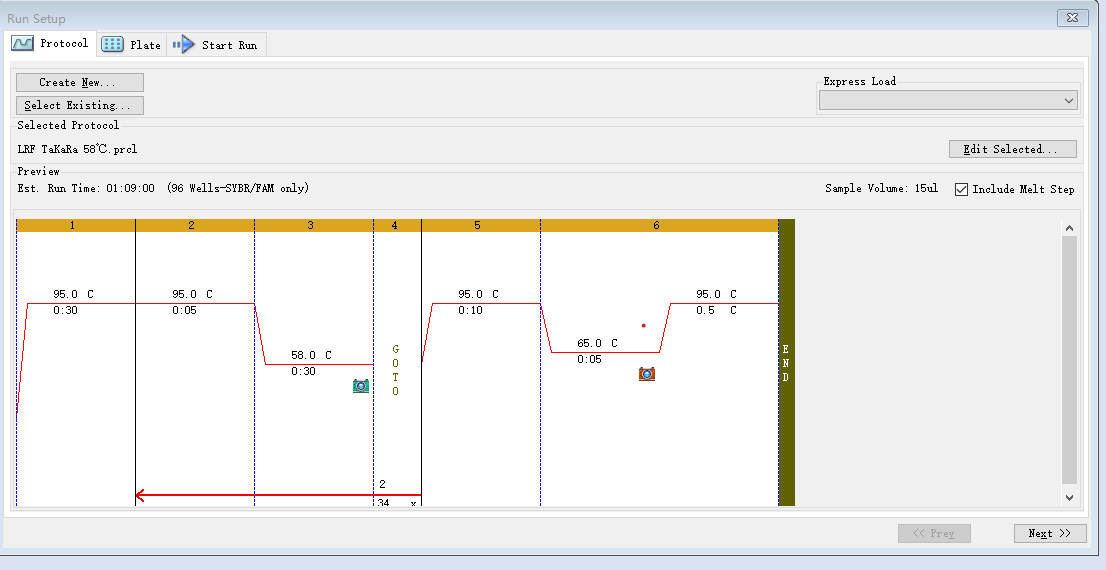


3 Primer information

Table S1 Primer sequences of genes encoding enzymes involved in 2-AP biosynthesis.

| Gene name | Accession no. | Primer sequences |
| --- | --- | --- |
| Proline dehydrogenase (ProDH) | AP014966.1 | F 5'-TCATCAGACGAGCAGAGGAGAACAGG-3' |
|  |  | R 5'-CCCAGCATTGCAGCCTTGAACC-3' |
| Pyrroline-5-carboxylic acid synthetase2 (P5CS2) | AP014957.1 | F 5'-GAGGTTGGCATAAGCACAG-3' |
|  |  | R 5'-CTCCCTTGTCGCCGTTC-3' |
| Ornithine aminotransferase (OAT) | AP014959.1 | F 5'-GCCCTTGGTGCTGGAGTA-3' |
|  |  | R 5'-AGCCCTTTCAACGAGACCTT-3' |
| Diamine oxidase2 (DAO2) | AP014960.1 | F 5'-TCGTTCGCATCAAGGTTGG-3' |
|  |  | R 5'-TCAGACAGAAGGGTGCCGTA-3' |
| Diamine oxidase4 (DAO4) | AP014960.1 | F 5'-TGGCAAGATAGAAGCAGAAGT-3' |
|  |  | R 5'-GTCCATACGGGCAACAAA-3' |
| Betaine aldehyde dehydrogenase (BADH2) | AB09683 | F 5'-GGTTGGTCTTCCTTCAGGTGTGC-3' |
|  |  | R 5'-CATCAACATCATCAAACACCACTAT-3' |
| Pyrroline-5-carboxylate reductase (P5CR) | AK067368 | F 5'-CGGGTAAACATCCAGGGCAGC-3' |
|  |  | R 5'-TTGTCACCATTCACCACTTGCCC-3' |
| OsActin | AK101613 | F 5'-CTTCATAGGAATGGAAGCTGCGGGTA-3' |
|  |  | R 5'-CGACCACCTTGATCTTCATGCTGCTA-3' |
